# Risk of pneumococcal bacteremia in Kenyan children with glucose-6-phosphate dehydrogenase deficiency

**DOI:** 10.1186/s12916-020-01604-y

**Published:** 2020-06-15

**Authors:** James J. Gilchrist, Sophie Uyoga, Matti Pirinen, Anna Rautanen, Salim Mwarumba, Patricia Njuguna, Neema Mturi, The Kenyan Bacteraemia Study Group, Adrian V. S. Hill, Thomas N. Williams, J. Anthony G. Scott, Stephen J. Chapman, Anna Rautanen, Tara C. Mills, Kirk Rockett, Anne W. Ndungu, Vivek Naranbhai, Alex W. Macharia, Sophie Uyoga, Carolyne Ndila, Neema Mturi, Patricia Njuguna, Shebe Mohammed, James A. Berkley, Isaiah Mwangi, Salim Mwarumba, Barnes S. Kitsao, Brett S. Lowe, Susan C. Morpeth, Iqbal Khandwalla, Herbert Opi, Emily Nyatichi, Prophet Ingosi, Barnes Kitsao, Clement Lewa, Johnstone Makale, Adan Mohamed, Kenneth Magua, Mary Njoroge, Gideon Nyutu, Ruth Mwarabu, Metrine Tendwa, Ismail Ahmed, Samuel Akech, Alexander Balo Makazi, Mohammed Bakari Hajj, Andrew Brent, Charles Chesaro, Hiza Dayo, Richard Idro, Patrick Kosgei, Kathryn Maitland, Kevin Marsh, Laura Mwalekwa, Shalton Mwaringa, Charles Newton, Mwanajuma Ngama, Allan Pamba, Norbert Peshu, Anna Seale, Alison Talbert, Adrian V. S. Hill, J. Anthony G. Scott, Thomas N. Williams

**Affiliations:** 1 Wellcome Centre for Human Genetics, University of Oxford, Oxford, OX3 7BN UK; 2 Department of Paediatrics, University of Oxford, Oxford, OX3 9DU UK; 3 KEMRI-Wellcome Trust Research Programme, Kilifi, 80108 Kenya; 4 Institute for Molecular Medicine Finland (FIMM), University of Helsinki, 00014 Helsinki, Finland; 5 The Jenner Institute, University of Oxford, Old Road Campus Research Building, Oxford, OX3 7DQ UK; 6 Department of Infectious Disease Epidemiology, London School of Hygiene & Tropical Medicine, Keppel Street, London, WC1E 7HT UK; 7 Department of Medicine, Imperial College, Norfolk Place, London, W2 1PG UK

**Keywords:** G6PD deficiency, Pneumococcus, Bacteremia, Malaria, Africa, Children

## Abstract

**Background:**

Glucose-6-phosphate dehydrogenase (G6PD) deficiency is the most common enzyme deficiency state in humans. The clinical phenotype is variable and includes asymptomatic individuals, episodic hemolysis induced by oxidative stress, and chronic hemolysis. G6PD deficiency is common in malaria-endemic regions, an observation hypothesized to be due to balancing selection at the G6PD locus driven by malaria. G6PD deficiency increases risk of severe malarial anemia, a key determinant of invasive bacterial disease in malaria-endemic settings. The pneumococcus is a leading cause of invasive bacterial infection and death in African children. The effect of G6PD deficiency on risk of pneumococcal disease is undefined. We hypothesized that G6PD deficiency increases pneumococcal disease risk and that this effect is dependent upon malaria.

**Methods:**

We performed a genetic case-control study of pneumococcal bacteremia in Kenyan children stratified across a period of falling malaria transmission between 1998 and 2010.

**Results:**

Four hundred twenty-nine Kenyan children with pneumococcal bacteremia and 2677 control children were included in the study. Among control children, G6PD deficiency, secondary to the rs1050828 G>A mutation, was common, with 11.2% (*n* = 301 of 2677) being hemi- or homozygotes and 33.3% (*n* = 442 of 1329) of girls being heterozygotes. We found that G6PD deficiency increased the risk of pneumococcal bacteremia, but only during a period of high malaria transmission (*P* = 0.014; OR 2.33, 95% CI 1.19–4.57). We estimate that the population attributable fraction of G6PD deficiency on risk of pneumococcal bacteremia in areas under high malaria transmission is 0.129.

**Conclusions:**

Our data demonstrate that G6PD deficiency increases risk of pneumococcal bacteremia in a manner dependent on malaria. At the population level, the impact of G6PD deficiency on invasive pneumococcal disease risk in malaria-endemic regions is substantial. Our study highlights the infection-associated morbidity and mortality conferred by G6PD deficiency in malaria-endemic settings and adds to our understanding of the potential indirect health benefits of improved malaria control.

## Background

Glucose-6-phosphate dehydrogenase (G6PD) catalyzes the oxidation of glucose-6-phosphate, producing 6-phosphogluconolactone and dihydronicotinamide adenine dinucleotide phosphate (NADPH) [1]. NADPH is a reducing agent that is central to the mechanism by which cells mitigate oxidative stress. This is especially important for erythrocytes, which, not possessing mitochondria, lack alternative metabolic pathways for NADPH synthesis. In humans, G6PD is encoded by an X-linked locus: *G6PD*.

*G6PD* is highly polymorphic. Deleterious *G6PD* polymorphisms result in the most common human enzyme deficiency: G6PD deficiency [1]. The X-linked inheritance of such polymorphisms means that G6PD deficiency is limited to hemizygous males and homozygous females, while heterozygous females display an intermediate phenotype. G6PD deficiency can cause a wide spectrum of clinical disease, the severity of which is dependent on the level of residual enzyme activity. In individuals with symptomatic G6PD deficiency, disease commonly manifests as episodic hemolysis induced by oxidative stress (e.g., drugs, infection). More severe defects cause chronic hemolysis, characterized by chronic transfusion-dependent anemia, splenomegaly, and the formation of gallstones [2]. An immunodeficiency characterized by granulocyte dysfunction and a clinical phenotype similar to chronic granulomatous disease has also been described in severe enzymatic deficiency [3]. The major genetic determinant of G6PD deficiency in coastal Kenyan populations is the rs1050828 G>A mutation [4], which gives rise to a form of G6PD deficiency that is commonly referred to as the G6PD A− variant. G6PD A− is a WHO class 3 G6PD deficiency variant [5], predicted to result in only mild-to-moderate enzymatic deficiency (10–60% of normal). This genetic variant of G6PD deficiency is estimated to account for 85% of phenotypic variation in G6PD enzymatic activity in coastal Kenyan populations [4]. Among female heterozygous for the G6PD A− variant in coastal Kenya, G6PD enzymatic activity is determined by variation at a second SNP at the G6PD locus, rs1050829 [4]. rs1050829 is a WHO class 4 G6PD deficiency variant, predicted to result in a clinically asymptomatic decrease in G6PD enzymatic activity (< 40% reduction from wild type). Variation at rs1050829 does not affect residual enzymatic activity in individuals with G6PD deficiency secondary to the G6PD A− variant in African populations, as the G6PD A− variant is always inherited on a rs1050829:C background [4].

The geographic distribution of G6PD deficiency is strongly correlated with malaria transmission. This has led to the hypothesis that malaria has driven selection of G6PD deficiency variants in human populations. That hypothesis is supported by evidence of recent selection pressure at *G6PD* [6] and by studies defining the effect of *G6PD* variation on malaria risk [7, 8]. The exact nature of the selection pressure imposed by malaria at *G6PD* remains controversial. We have previously demonstrated that, in Kenyan children, G6PD status is not associated with uncomplicated malaria, but that G6PD deficiency secondary to the G6PD A− variant increases the risk of severe malarial anemia (SMA), whereas heterozygous females are protected against severe malaria [7]. In the same population, we found no evidence for an independent effect of C allele carriage at rs1050829 on risk of uncomplicated or severe malaria [7]. Furthermore, through a multi-population analysis of the effect of variation at *G6PD* on malaria risk, we have recently demonstrated that G6PD deficiency is associated with opposing additive effects on cerebral malaria and SMA [8]. In that analysis, G6PD deficiency was associated with increased susceptibility to SMA but a lower risk of cerebral malaria [8]. More recently, however, it has been suggested that this observation may represent an artifact of collider bias [9]: by excluding children with severe anemia from the case definition of cerebral malaria, the apparent protective association may be driven by an absence of severe anemia and not cerebral malaria per se*.* Notwithstanding on-going uncertainty regarding the exact nature of the balancing selection at the G6PD locus driven by malaria, there is a clear and reproducible association between G6PD deficiency and risk of SMA.

The effect of genetic variation at *G6PD* on infectious diseases other than malaria has not been widely studied. Specifically, the role for genetic variation at the *G6PD* locus as a determinant of invasive bacterial disease is undefined. *Streptococcus pneumoniae* is consistently among the most frequently isolated pathogens causing bacteremia in African children [10] and is a leading cause of mortality in children < 5 years [11]. Genetic variation at *G6PD* modifies risk of SMA. SMA is a major risk factor for community-acquired bacteremia in African children [12, 13], and the malaria-protective effects of HbAS carriage reduce the risk of some of the clinical syndromes that are associated with invasive pneumococcal diseases such as severe pneumonia and meningitis [14]. Moreover, severe anemia in African children, irrespective of its etiology, is associated with increased risk of community-acquired bacteremia [15]. We thus hypothesized that G6PD deficiency increases the risk of pneumococcal bacteremia in African children and that this effect is dependent on intense malaria transmission resulting in severe anemia among G6PD-deficient children. To test this hypothesis, we performed a genetic case-control analysis of the effect of G6PD deficiency on the risk of pneumococcal bacteremia in Kenyan children, across a period of falling malaria transmission.

## Methods

### Study population, genotyping, and quality control

Kenyan children under the age of 13 years, presenting to Kilifi County Hospital, Kenya, between 1 August 1998 and 30 October 2010, with community-acquired bacteremia were recruited to the study. Routine vaccination against *S. pneumoniae* was not available during the study period and was not introduced until January 2011. During the study period, a blood sample for bacterial culture was obtained from every child admitted to hospital, with the exception of elective surgical admissions and children admitted with trauma. Bacterial culture of blood was performed with a BACTEC 9050 instrument (Becton Dickinson, USA), and pneumococcal isolates identified by optochin susceptibility. Children with a mid-upper arm circumference < 11.5 cm (< 11 cm in children < 6 months of age) were considered to have severe malnutrition. HIV infection status was determined with two rapid antibody tests and with PCR for proviral DNA for children < 18 months. Malaria parasitemia was assessed using thick and thin Giemsa-stained blood films. Among children with all-cause bacteremia (*n* = 1816), 506 cases of pneumococcal bacteremia were recruited to the study. Healthy community controls (*n* = 3091) were recruited as part of a birth cohort study at 3–12 months of age from within the same population as cases between 1 May 2006 and 30 April 2008. All children have been subject to longitudinal follow-up.

Genome-wide genotyping was performed in all study samples as part of the Wellcome Trust Case Control Consortium 2 genome-wide association study (GWAS) of all-cause bacteremia [16]. Following genomic DNA extraction and quality control, DNA was whole-genome genotyped on Affymetrix SNP Chip 6.0 arrays. SNPs passing the following quality control (QC) metrics (MAF > 1%, genotype probability (info) > 0.975, plate effect *P* > 1 × 10^−6^, Hardy-Weinberg equilibrium *P* < 1 × 10^−10^, and SNP missingness > 2%) were included in the analysis. Following SNP QC, genotypes at 787,861 autosomal SNPs were taken forward for computation of principal components (EIGENSTRAT [17]) following linkage disequilibrium pruning, relatedness estimates (identity by descent, PLINK [18]) and heterozygosity (PLINK). Samples were then excluded from the association analysis as follows: relatedness (identity by descent > 0.4), population outliers, extreme heterozygosity, or low genotyping call rate [16].

Genotypes at rs1050828 were directly determined on the genotyping array. Genotyping quality at rs1050828 was good, passing SNP QC thresholds (as above) and with good cluster separation (visualized in Evoker). Genotypes at rs1050829 and the 4 malaria risk loci (*ABO*, rs8176719; Dantu blood group, rs192804806; sickle hemoglobin [HbS], rs334; *ATP2B4*, rs4951377) were not directly determined and were imputed with SHAPEIT [19] and IMPUTE2 [20], using 1000G phase 3 as a reference panel. Following quality control, 429 cases of *S. pneumoniae* bacteremia and 2677 healthy control samples were included in the association analysis.

### Statistical analysis

#### Association analysis at G6PD

We used logistic regression to test for association between pneumococcal bacteremia and G6PD A− variant (rs1050828) genotype under additive (rs1050828 genotypes coded to reflect a monotonic change in G6PD biochemical activity), G6PD deficiency (G6PD deficiency c.f. normal G6PD activity), and G6PD heterozygous (rs1050828:T heterozygous girls c.f. normal G6PD activity) models. In a separate model, we used logistic regression to test for association between pneumococcal bacteremia and rs1050829 genotype among G6PD A− heterozygous females under an additive model. The first four principal components of genome-wide genotyping data were included in each model to account for population sub-structure. In addition, sex was included as a covariate in each model. Statistical analysis was performed in R.

#### Multinomial logistic regression

As control samples were recruited as a birth cohort towards the end of the study, we used multinomial logistic regression to estimate the effect of the G6PD variation on pneumococcal bacteremia risk in each of the three phases of malaria transmission (pre-decline, pre-2000; decline, 2000–2006; post-decline, post-2006). We used control status as the baseline stratum and cases of pneumococcal bacteremia during each of the specified time periods as strata. We tested for association under additive, G6PD deficiency, and G6PD heterozygous models (as defined above) at rs1050828, and the effect of rs1050829 genotype in rs1050828 heterozygous girls under an additive model. In each case, the first four principal components and sex were included as covariates in the model. To address the possibility of confounding secondary to other known malaria risk loci, we also included genotypes at each locus in the model, with genotypes coded to reflect the model of association observed in malaria (*ABO*, recessive; Dantu blood group, additive; HbS, heterozygote and recessive; *ATP2B4*, recessive).

To identify a subset of control samples well-matched to cases with respect to sex and ethnicity (as modeled by the first 4 principal components of genome-wide genotyping data), we used nearest neighbor matching as implemented in MatchIt [21]. The proportions of case children with HIV infection and malnutrition were compared between time periods using *χ*^2^ tests. Under the assumption that G6PD deficiency and HIV infection or malnutrition are independent, we performed case-only interaction analysis with logistic regression adjusted for sex and population structure. Statistical analysis was performed in R.

#### Bayesian comparison of models of association

We compared models of association at rs1050828 with pneumococcal bacteremia across three time periods, as estimated by multinomial logistic regression, using a Bayesian approach. We considered three models of effect across the specified time periods defined by the prior distributions on the effect size:

- “Null”: effect size = 0, i.e., no association in any time period.
- “Same”: effect size ~ *N* (0,*a*^2^) and fixed between time periods (*ρ* = 1).
- “Pre-decline alone”: effect size ~ *N* (0,*a*^2^) in the pre-decline period only (with no effect in the other time periods).

For heterozygous and G6PD deficiency models, *a* = 0.5, and for additive models, *a* = 0.2. For each model, we calculated approximate Bayes factors [22] and posterior probabilities, assuming each model to be equally likely a priori. Statistical analysis was performed in R.

#### Population attributable fraction

Population attributable fractions were calculated as follows:

$$ \mathrm{PAF}=\frac{P\left(\mathrm{OR}-1\right)}{P\left(\mathrm{OR}-1\right)+1} $$

where PAF = population attributable fraction, OR = odds ratio, and *P* = population frequency of risk genotype(s).

## Results

### Study population

**Fig. 1:**
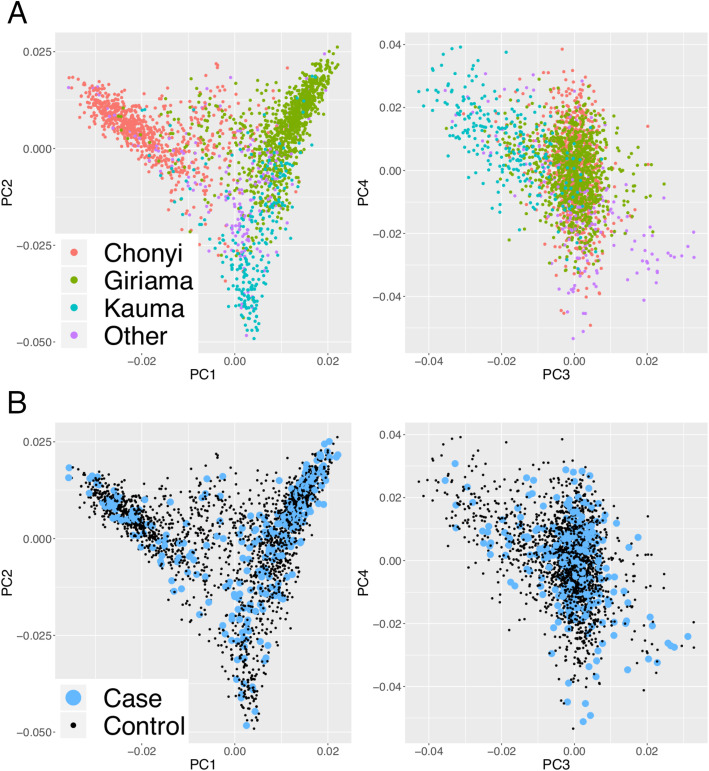
Population structure of study samples. Plots of the first four principal components of genome-wide genotyping data. Individuals are color-coded according to self-reported ethnicity (**a**) and case-control status (**b**)

Following quality control, 429 Kenyan children with pneumococcal bacteremia and 2677 control participants were included in our genetic association analysis. The first four principal components of genome-wide genotyping data capture self-reported ethnicity and confirm that the population structure of the control samples is representative of the cases (Fig. 1). The mean age among case samples was 1.9 years (range 0–13 years), and 38.7% were female. Among cases, at admission, 25.9% had malnutrition, 12.2% had malaria parasitemia, and 20.8% were HIV-infected (Table 1). Inpatient mortality was 24%. The use of a birth cohort as a healthy control population risks loss of study power through misclassification bias: the inclusion of children as healthy controls who have subsequently experienced an episode of pneumococcal bacteremia. Follow-up of the control children demonstrates that the effect of misclassification bias in our study is negligible. Following longitudinal follow-up to an average age of 5.1 years, there were 8 cases of all-cause bacteremia and 24 deaths among the control children included in the study.

**Table 1:** Study sample demographics, case comorbidities, and inpatient mortality

		Cases	Controls
	Median age in months (range)	23 (0–157)	*
	Females, *n* (%)	166 (38.7)	1329 (49.6)
Reported ethnicity	Giriama, *n* (%)	167 (38.9)	1226 (45.8)
	Chonyi, *n* (%)	78 (18.2)	993 (37.1)
	Kauma, *n* (%)	22 (5.1)	318 (11.9)
Comorbidities	HIV-infected, *n* (%)**	25 (20.8)	NA
	Malnutrition, *n* (%)	108 (25.9)	NA
	Malaria, *n* (%)	44 (12.2)	NA
	Mortality, *n* (%)	101 (24.0)	NA

*NA* not applicable

*Birth cohort

**HIV-status ascertained in subset of cases

### Genetic variation causing G6PD deficiency

Among control samples, rs1050828 had a minor allele frequency (MAF) of 0.196. G6PD deficiency, defined by homozygous rs1050828:AA or hemizygous rs1050828:A genotypes, was common (*n* = 301 of 2677; 11.2%). 3.2% of girls (*n* = 42 of 1329) and 19.2% of boys (*n* = 259 of 1348) had homozygous and hemizygous A allele carriage at rs1050828, respectively, resulting in G6PD deficiency. One third (33.3%, *n* = 442 of 1329) of girls were heterozygous for rs1050828:A carriage. rs1050829 was well-imputed and common in this dataset (imputation info metric = 0.981; MAF = 0.396). In the study population, 27.9% (*n* = 1175 of 4213) were homozygous or hemizygous for the minor rs1050829:C allele. Among female rs1050828:T heterozygotes, 74.9% (*n* = 490 of 654) were rs1050829:TC heterozygotes and 25.1% (*n* = 164 of 654) were rs1050829:CC homozygotes. The 4 malaria risk loci previously described in this population [23] were common and well-imputed among the study samples (rs8176719, imputation info metric = 0.994, MAF = 0.259; rs334, imputation info metric = 0.932, MAF = 0.097; rs192804806, imputation info metric = 0.987, MAF = 0.092; rs4951377, imputation info metric = 0.995, MAF = 0.658).

### G6PD status and risk of pneumococcal bacteremia

To investigate whether G6PD status is a determinant of pneumococcal bacteremia risk, we tested for association between the rs1050828 locus and pneumococcal bacteremia. Genotype at rs1050828 (*P*_additive_ = 0.261; OR = 1.08, 95% CI 0.94–1.25), G6PD deficiency (*P* = 0.080; OR = 1.30, 95% CI 0.96–1.74), and heterozygous carriage of the rs1050828:A allele (*P* = 0.326; OR = 0.83, 95% CI 0.56–1.19) were not associated with risk of pneumococcal bacteremia. In addition, among female rs1050829 G>A heterozygotes, rs1050829 carriage did not modify risk of pneumococcal bacteremia (*P*_additive_ = 0.452; OR = 0.75, 95% CI 0.36–1.58).

### G6PD deficiency increases risk of pneumococcal bacteremia during an era of high malaria transmission

**Fig. 2:**
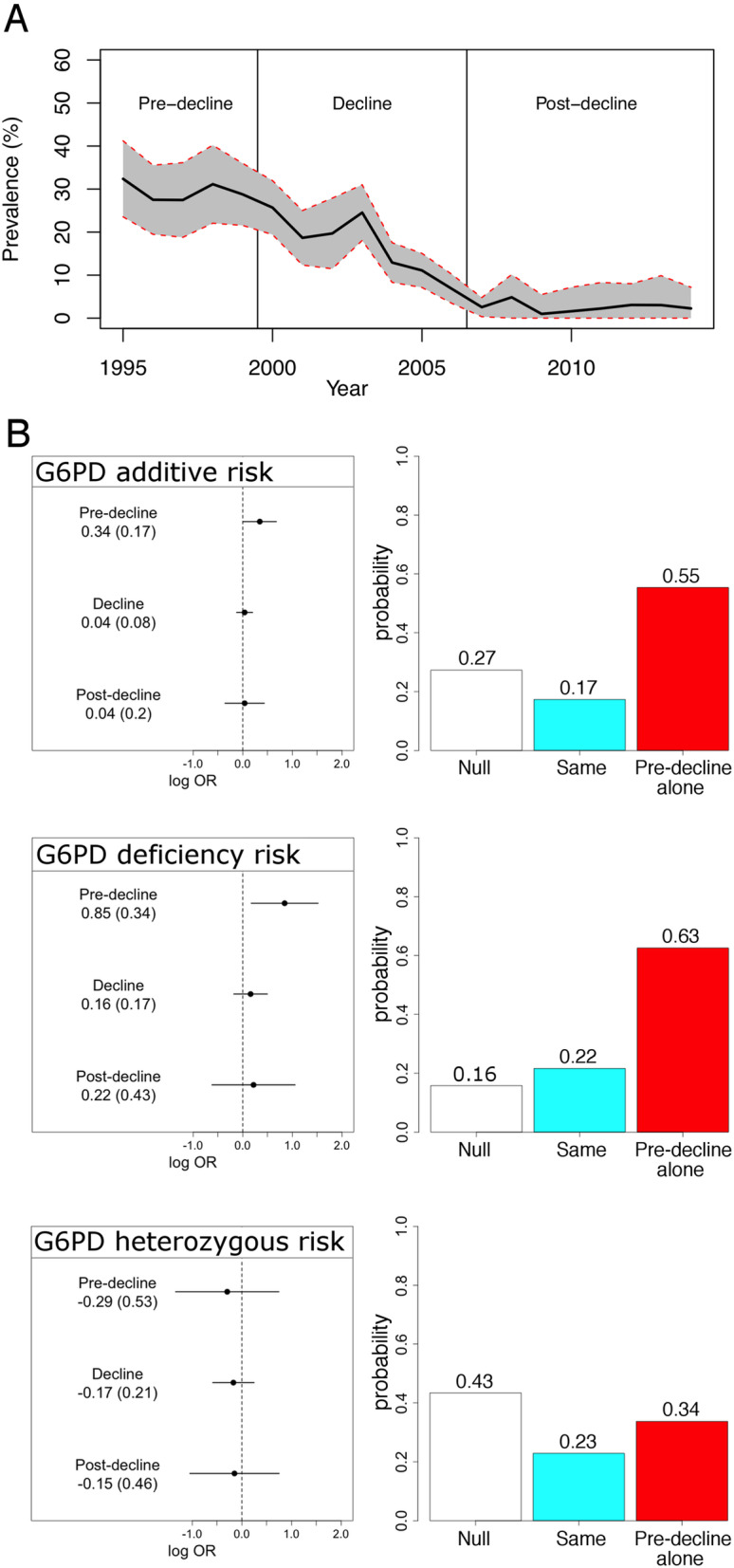
Malaria transmission, G6PD deficiency, and risk of pneumococcal bacteremia. **a** Age-standardized, annual malaria parasite prevalence in Kilifi, Kenya, as estimated from parasite prevalence among trauma admissions [24, 25]. Ninety-five percent confidence intervals illustrated with red, dashed line. Pre-decline (pre-2000), decline (2000–2006), and post-decline (post-2006) periods used in the analysis are depicted. **b** Left panels: Log-transformed odds ratios and 95% confidence intervals of G6PD deficiency association with pneumococcal bacteremia risk in pre-decline, decline, and post-decline study periods. Right panels: Posterior probabilities of models of association with G6PD deficiency: “null,” no association with pneumococcal bacteremia in any time period; “same,” the same effect on bacteremia in all three time periods; and “pre-decline alone,” a non-zero effect on pneumococcal bacteremia in the pre-decline time period alone. Association statistics and model posterior probabilities are presented under additive (top), G6PD deficiency risk (middle), and G6PD heterozygous risk (bottom) models

Over the course of case recruitment, between 1998 and 2008, there was a marked change in malaria transmission intensity in Kilifi [24]. Historically, malaria has been a major risk factor for community-acquired bacteremia in this population [13]. Declining malaria transmission may therefore confound any association between bacteremia and G6PD deficiency. To investigate this, we estimated the effect of G6PD status on pneumococcal bacteremia risk as malaria transmission declined over the period of the study. Dividing the study period into pre-decline (pre-2000), decline (2000–2006), and post-decline (post-2006) periods, we fitted multinomial regression models of pneumococcal bacteremia risk secondary to G6PD status, considering each time period as a stratum (Fig. 2). Under an additive model, the data provide support for a model in which decreasing G6PD activity (estimated by rs1050828 genotype) only increased the risk of pneumococcal bacteremia during the pre-decline period (OR_Pre-decline_ = 1.41, 95% CI 1.01–1.97, *P*_Pre-decline_ = 0.044). However, the data more strongly support a model in which G6PD deficiency, defined by homozygous rs1050828:AA or hemizygous rs1050828:A genotypes, increased risk of pneumococcal bacteremia, again in the pre-decline period alone (OR_Pre-decline_ = 2.33, 95% CI 1.19–4.57, *P*_Pre-decline_ = 0.014). We found no evidence for a significant effect of heterozygous carriage of rs1050828:A on pneumococcal bacteremia risk in any time period. We saw no evidence for a significant effect of rs1050829:C carriage on pneumococcal bacteremia risk in any time period.

To ensure that our model adequately controls for differences in sex and population structure, we used propensity score matching to identify a subset of control samples (*n* = 429) matched to cases for sex and population structure. In keeping with our primary analysis, there is again evidence for increased risk of pneumococcal disease among children with G6PD deficiency (OR_Pre-decline_ = 2.54, 95% CI 1.23–5.25, *P*_Pre-decline_ = 0.012). There is no evidence for effect of G6PD deficiency on pneumococcal disease risk in the decline and post-decline study periods (*P*_Decline_ = 0.271, *P*_Post-decline_ = 0.442).

### G6PD deficiency increases risk of pneumococcal bacteremia independent of other risk factors for invasive infection

Both HIV infection and malnutrition are established risk factors for pneumococcal bacteremia in this population [26]. We therefore sought to understand whether changes in the epidemiology of malnutrition or HIV infection during the study could confound our findings. We compared the proportions of cases with HIV co-infection and malnutrition presenting during the pre-decline period and during later years. There was evidence for a temporal change in the proportion of cases with malnutrition (pre-2000, 12 of 47 children, 25.5%; post-2000, 86 of 370 children, 23.2%; *P* = 5.13 × 10^−4^), but not with HIV infection (pre-2000, 11 of 44 children, 25%; post-2000, 14 of 86 children, 16.3%; *P* = 0.233). There was no evidence for interaction between G6PD deficiency and malnutrition or HIV among cases of pneumococcal bacteremia in the study considered as a whole (*P*_Malnutrition_ = 0.92; *P*_HIV_ = 0.77) or during the pre-decline period alone (*P*_Malnutrition_ = 0.85; *P*_HIV_ = 0.48).

Genome-wide association studies have identified 4 genetic determinants of severe malaria risk in this population (ABO blood group, HbS, Dantu blood group, and *ATP2B4*) [23]. We investigated whether the observed association between G6PD deficiency and the risk of pneumococcal bacteremia was independent of genetic variation at these loci. Including genetic variation at these loci did not significantly alter the effect estimate of G6PD deficiency on pneumococcal bacteremia risk in the study considered as a whole (OR = 1.28, 95% CI 0.95–1.73, *P* = 0.101) or during the pre-decline period alone (OR = 2.32, 95% CI 1.18–4.56, *P* = 0.015).

### The population attributable fraction of pneumococcal bacteremia

To better understand the impact of G6PD deficiency on pneumococcal disease risk, we calculated the population attributable fraction prior to the observed decline in malaria transmission at the study site. In the pre-decline period, the pneumococcal bacteremia population attributable fraction for G6PD deficiency was 0.129. For comparison, assuming an OR for pneumococcal bacteremia among children with sickle cell disease (HbSS) of 33.0 [27] and a mean HbSS frequency among children < 14 years in this population of 0.003 [27], the population attributable fraction of pneumococcal bacteremia for HbSS would be 0.088.

## Discussion

In Kenyan children, G6PD deficiency increases risk of pneumococcal bacteremia. This effect is only observed prior to a decline in malaria transmission at the study site. Heterozygous carriage of the rs1050828:A allele does not significantly affect risk of pneumococcal bacteremia in this population. These data demonstrate that G6PD deficiency increases risk of pneumococcal bacteremia in Kenyan children and that this effect is dependent on malaria.

Malaria is a well-established risk factor for community-acquired bacteremia [13]. Children with G6PD deficiency secondary to the G6PD A− variant have increased risk of SMA in this population [7]. Epidemiological [28] and immunological [29] data have defined a role for SMA in susceptibility to invasive bacterial disease, including pneumococcal bacteremia. In keeping with this, we observed a malaria-dependent increase in risk of pneumococcal bacteremia among children with G6PD deficiency secondary to the G6PD A− variant. By contrast, carriage of the rs1050829:C allele, a WHO class 4 deficiency variant, was not associated with increased risk of SMA in Kenyan children [7] and was not associated with increased risk of pneumococcal bacteremia in this study. This effect may therefore reflect an increased risk of SMA among G6PD-deficient children. It is important to note, however, that the increased risk of malaria-induced hemolysis conferred by G6PD deficiency will result in anemia and immunological deficits that will persist beyond the index malaria infection. Indeed, in this population, recent malaria infection is a stronger predictor of nontyphoidal *Salmonella* bacteremia than concurrent malaria [30].

We did not see any protective effect of heterozygosity at *G6PD* on pneumococcal bacteremia risk. Bacteremia complicating cerebral malaria is uncommon. If the malaria-protective effects of G6PD deficiency heterozygosity are restricted to cerebral malaria, these effects would not be predicted to modify invasive bacterial disease risk, consistent with our observations. That we do not observe a protective effect of G6PD deficiency heterozygosity on pneumococcal disease risk may suggest that G6PD deficiency alleles do indeed confer specific protection against cerebral malaria, but may equally represent a lack of study power to detect such an effect. Future studies are needed to clarify protective effects of G6PD deficiency heterozygosity on malaria and bacteremia risk.

Malaria control interventions have effects on mortality among African children in excess of those attributable to the observed reduction in severe malaria syndromes [31, 32]. In this study, G6PD deficiency was estimated to account for 13% of cases of pneumococcal bacteremia, but only during a period of high malaria transmission. This highlights the significant risk of morbidity and mortality secondary to invasive pneumococcal disease conferred by G6PD deficiency in malaria-endemic settings. These data also highlight that health outcomes for children with G6PD deficiency will be improved by better malaria control interventions and that these benefits will be both directly and indirectly attributable to improved malaria control.

## Conclusions

Our data define a role for G6PD deficiency in susceptibility to pneumococcal bacteremia in Kenyan children. We propose a model in which G6PD deficiency increases risk of malaria-induced anemia in Kenyan children, thereby increasing risk of invasive pneumococcal disease. Our data highlight the importance of G6PD deficiency as a determinant of susceptibility to infection and further highlights the potential indirect benefits of improved malaria control in malaria-endemic populations.

## Data Availability

Genotype and phenotype data are available via the European Genotype Archive, with the accession code EGAD00010000950.

## Abbreviations

G6PD: Glucose-6-phosphate dehydrogenase
HbS: Sickle cell hemoglobin
NADPH: Dihydronicotinamide adenine dinucleotide phosphate
PAF: Population attributable fraction
SMA: Severe malarial anemia
